# 

**Published:** 1897-12-25

**Authors:** 


**Notices of Births, Deaths, and Marriages are inserted in this column at a charge of 2s. 6d. for an announcement not exceeding four lines.**

### NOTICE TO CORRESPONDENTS.

**All MS., Letters, Books for Review, and other matters intended for the Editor should be addressed The Editor, "The Hospital," The Hospital Building, 28 & 29, Southampton Street, Strand, W.C.**

**The Editor cannot undertake to return rejected MS., even when accompanied by stamped directed envelope.**

**Notice to Advertisers and Subscribers.**

**All Advertisements, Orders for Copies of The Hospital, and Business Communications should be addressed to THE MANAGER, (Not to The Editor), The Hospital, The Hospital Building, 28 & 29, Southampton Street, Strand, London, W.C.**

**The Cost of Subscription, post free, is as follows:—Per week, 2½d.; per quarter, 2s. 8d.; per half-year, 5s. 3d.; per annum, 10s. 6d. Foreign:—Per annum, 15s. 2d.; payable, in all cases, in advance.**

**NOTICE.—Vol. XXII. of "The Hospital" (April, 1897, to September, 1897), in handsome cloth binding, is now on sale at the Office of this Journal, price 7s. 6d. Cases for binding the Numbers contained in this Volume, 1s. 6d. Index to Vol. XXII., 2½d., post free. The Monthly Part for January will be ready on January 1st, Price 6d., by post 8d.**

### BOOKS RECEIVED.

Macmillan and Co.
"Transactions of the British Institute of Preventive Medicine."

G. P. Putnams' Sons.
"Ambroise Paré and His Times." By Stephen Paget.

The Roxburghe Press.
"Facts about Monte Carlo."

### Scraps and Gleanings.

The average cost per patient per week at the Jamaica Lunatic Asylum in 1896-7 was 6s. 10½d.

The Worshipful Company of Skinners have forwarded a further donation of £21 to the British Home for Incurables at Streatham.

The expenditure of 1896 at the South Australian asylums was £21,459, or £776 less than for 1895, although 20 more patients were treated. The daily average cost per head was 1s. 3$\frac{1}{10}$d.

The Queen, who has for the past 50 years been patron of the Royal Hospital for Diseases of the Chest in the City Road, has sent 70 lbs. of cast linen for the patients.

The Salters' Company have granted £26 5s. to the Children's Branch Building Fund, and the Cordwainers' Company £21 to the general fund of the Metropolitan Convalescent Institution.

Among recent contributions towards the endowment of the "Princess Mary" bed at the Chelsea Hospital for Women, Fulham Road, S.W., have been £105 from Mr. Matthew Dobson, £25 from Miss S. Brisco, and £21 from the Skinners' Company.

Of the 573 in-patients admitted in 1896-7 to the Victorian Eye and Ear Infirmary, at Melbourne, 23 per cent. resided in the colony outside a radius of 50 miles from Melbourne, and of the 4,148 out-patients nearly 12 per cent. came from the same district.

The number of insane persons remaining in the two lunatic asylums of South Australia on the last day of 1896 was 934, viz., 540 males and 394 females. This makes an increase of four on the number on December 31st, 1895. The total number treated was 1,125, against 1,105 in 1896.

In the annual report of the Jamaica Lunatic Asylum for the year ended March 31st, 1897, attention is called to the increase in the number of the inmates of the institution. The year began with 664 patients; it ended with 722; and it seems probable that every year for some years to come 50 additional beds will have to be provided and maintained.

The number of cases of lunacy under treatment in the five lunatic asylums for natives in the Bengal Presidency during the years 1895 and 1896 are curiously similar. Thus, on January 1st, 1895, there were 923 cases in the asylums, and 184 were admitted during the year; while on January 1st, 1896, there were 921 cases under treatment, and 181 were admitted. The daily average strength was 919 and 914 respectively.

That there is much over-crowding at the Jamaica Lunatic Asylum is evidenced by the fact that the buildings now occupied are intended to accommodate about 550 cases, and they now house 722. New buildings are in progress of erection, and will give accommodation for 420 more patients, but it is anticipated that by the time the new buildings are ready for occupation—about the end of the next financial year—there will be but little reserve accommodation.

The National Refuges for Homeless and Destitute Children, 164, Shaftesbury Avenue, have just received a cheque for five guineas from the Worshipful Company of Salters, being the thirty-fourth annual subscription of the company to that charity. It is interesting to note that during the past fifty-four years these Refuges and their useful branches, the training ships "Arethusa" and "Chichester" have received over 14,000 destitute children. A grant of one hundred guineas has also been received from the Corporation of the City of London. This makes £1,445 contributed by the Corporation to these Refuges during the past twenty-one years

### The Student of Medicine.

### APPOINTMENTS.

B. Anningson, B.A.Cantab, M.R.C.S., appointed an Examiner for the Diploma of Public Health at the University of Cambridge. J. Buchanan, M.B., C.M.Edin., has been appointed Medical Officer for the Poor law Union of Watford. J. J. Callanan, L.R.C.P. & L.R.C.S & L M.Irel. appointed Medical Officer for Ribchester Workhouse, and Medical Officer and Public Vaccinator for the No. 3 District of the Preston Union. J. A. Clark, appointed Junior Assistant Medical Officer to the Glasgow City Parish Poorhouse. W. Collingridge, B.A Cantab., M.D., D.P.H., appointed an Examiner for the Diploma of Public Health at the University of Cambridge. G. Dickson, L.R.C.P.E., L.R.C.S.E., L.F.P.S.G., appointed Medical Officer of Health to the town of Hay, and Medical Officer for the Radnorshire District of the Hay Union. J. McPhail Dougall, M.D., M.B., C.M.Glas., appointed Medical Officer to the Castle Howard Reformatory. P. Dunn, F.R.C.S., appointed Ophthalmic Surgeon to the West London Hospital. H. FitzGibbon, M.D., T.C.D., M.Ch., M.R.C.P.I., appointed Consulting Surgeon to the Mercer's Hospital, Dublin. W. E. Fry, M.R.C.S., L.R.C.P.Lond., appointed Medical Officer for the 4A District of the Malling Union. J. H. R. Glenn, B.A.Dub., M.B., appointed Gynæcologist to the Mercer's Hospital, Dublin. H. M. W. Gray, M.B., C.M.Aberd., appointed Assistant Anæsthetist to the Aberdeen Royal Infirmary. J. C. Harcourt, L.R.C.P., M.R.C.S., appointed Assistant Medical Officer to the Infirmary of the Lambeth Union. H. Holmes, B.A., M.B., B.C.Cantab., appointed Junior House Surgeon to the Royal Albert Edward Infirmary, Wigan. A. J. Hull, M.R.C.S.E., L.R.C.P., L.S.A., appointed Clinical Assistant to the Hospital for Women, Soho Square. H. T. Jones, L.R.C.P.I., M.R.C.S.Eng., appointed Medical Officer of Health to Hucknall-Torkard District Council. J. W. Jones, L.R.C.P., L.R.C.S.Edin., appointed Medical Officer and Public Vaccinator for No. 2 District of the Chorlton Union. A. A. Kanthack, M.D.Lond., B.Sc., F.R.C.S.Eng., appointed an Examiner for the Diploma of Public Health at the University of Cambridge. E. Le Geyt, M.R.C.S.Eng., L.R.C.P.Lond., appointed Resident Surgical Officer to the Infirmary, Birmingham. J. R. Levack, M.B., C.M.Aberd., appointed Medical Electrician to the Aberdeen Royal Infirmary. W. D. McMurtry, L.R.C.S.&P. Edin., appointed House Surgeon to the Memorial Hospital, Jarrow-on-Tyne. S. Martin, M.D., F.R.C.P., B.Sc.Lond., appointed Examiner in Pathology to the Victoria University. J. H. Moorhead, B.A., M.B., B.Ch.Durh., appointed Resident Medical Officer to the Suffolk General Hospital. C. P. Moreton, M.R.C.S.Eng., L.S.A., appointed Medical Officer of Health to the Forden Rural District Council. Dr. Muir, appointed Medical Officer for the Abertillery District of the Bedwelty Union. J. L. Notter, B.A.Dub., M.D., appointed an Examiner for the Diploma of Public Health at the University of Cambridge. G. W. Pickering, F.R.C.P., L.R.C.S.Edin., appointed Medical Officer for the Fourth District of the Malling Union. W. B. Pritchard, M.R.C.S., L.R.C.P.Lond., appointed Medical Officer for the No. 2 (St. Luke's Ward) District of the Chorlton Union. G. A. Reid, M.B., C.M.Aberd., appointed House Surgeon to the Royal Victoria Hospital, Bournemouth. G. A. G. Simpson, M.R.C.S.Eng., L.S.A., appointed Medical Officer of Health to Acton Urban District Council.

### PASS LIST.

### University of London

M.B. EXAMINATION: OCTOBER, 1897.

EXAMINATION FOR HONOURS.

Obstetric Medicine.—First Class: C. W. Alford, Middlesex Hospital; T. V. Cunliffe (gold medal), Owens College and Manchester Royal Infirmary; B. Dyball, St. Thomas's Hospital; W. T. Milton, Guy's Hospital; L. A. Smith, London Hospital; E. J. Toye (scholarship and gold medal), St. Bartholomew's Hospital. Second Class: A. Burn, St. Mary's Hospital; M. Dixon, University College; F. W. Robertson, St. Bartholomew's Hospital; A. W. Sikes, St. Thomas's Hospital. Third Class: W. A. Dewhirst, Yorkshire College; Mary Nona Sharman, Royal Free Hospital.

Forensic Medicine.—First Class: M. Dixon, (scholarship and gold medal), University College; C. H. Melland, Owens College and Manchester Royal Infirmary; W. T. Milton, Guy's Hospital; A. W. Sikes (gold medal), St. Thomas's Hospital; H. J. Starling, Guy's Hospital. Second Class: C. H. Fagge, Guy's Hospital; R. W. Mayston, Guy's Hospital; G. E. Richmond, B.A., B.Sc., Guy's Hospital; F. W. Robertson, St. Bartholomew's Hospital; L. A. Smith, London Hospital. Third Class: E. W. Adams, University College, Sheffield; T. V. Cunliffe, Owens College and Manchester Royal Infirmary; C. P. Harris, London Hospital; R. W. C. Pierce, B.Sc., St. Thomas's Hospital; Mary Nona Sharman, Royal Free Hospital; F. H. Thiele, University College; A. G. Wilson, London Hospital.

### University of Cambridge.

THIRD EXAMINATION FOR MEDICAL AND SURGICAL DEGREES.

MICHAELMAS TERM, 1897.

The following were examined and approved: Ambrose, Brincker, Carsberg, Cayley, Collingwood, Corner, Delbruck, Hunt, Johnson, Le Fleming, Levick, Lidderdale, Myers, Peatling, Percival, Reissmann, Sandilands, Sargent, Tucker.

